# Uncommon Cause of Pulmonary Hypertension: Imaging Diagnosis of Cardiac Myxoma Embolism

**DOI:** 10.3390/diagnostics15192420

**Published:** 2025-09-23

**Authors:** Alexandra Braillon, Paul Patural, David Laville, Frédéric Perros, Ségolène Turquier, Vincent Cottin, Romain L’Huillier, Salim Si-Mohamed

**Affiliations:** 1 Department of Medical Imaging, Louis Pradel Hospital, Hospices Civils de Lyon, 69002 Lyon, France; alexandra.braillon01@chu-lyon.fr; 2 Department of Medical Imaging, Pierre Wertheimer Hospital, Hospices Civils de Lyon, 69002 Lyon, France; paul.patural@chu-lyon.fr; 3 Pathology Department, CHU Louis Pradel, Hospices Civils de Lyon, 69002 Lyon, France; 4 Laboratoire CarMeN, UMR INSERM U1060/INRA U1397, Claude Bernard Lyon 1 University, 69500 Bron, France; 5 Pneumology Department, Hospices Civils de Lyon, National Reference Center for Rare Pulmonary Diseases, Constitutive Reference Center for Severe Pulmonary Hypertension, UMR 754 INRAE, ERN-LUNG, 69500 Bron, France; 6 Department of Medical Imaging, Edouard Herriot Hospital, Hospices Civils de Lyon, University of Lyon, 69002 Lyon, France; romain.lhuillier@chu-lyon.fr; 7 LabTAU, INSERM U1032, 69003 Lyon, France; romain.lhuillier@chu-lyon.fr; 8 Claude Bernard Lyon 1 University, INSA-Lyon, UJM-Saint Etienne, CNRS, Inserm, CREATIS UMR 5220, U1206, 69621 Lyon, France; salim.si-mohamed@chu-lyon.fr

**Keywords:** pulmonary hypertension, right atrial myxoma, endoluminal cell proliferation, arterial pulmonary aneurysm

## Abstract

We report an original case of pulmonary hypertension with artery aneurysms due to the cell proliferation of a right atrial myxoma with multi-modality imaging. Only three cases have been reported in the literature. The description of endoluminal cells proliferation in pulmonary arteries is rare on imaging, and this observation could be very useful in demonstrating not only the usefulness of multi-modality imaging, but also the combined performance of the dual-energy scanner.

**Figure 1:**
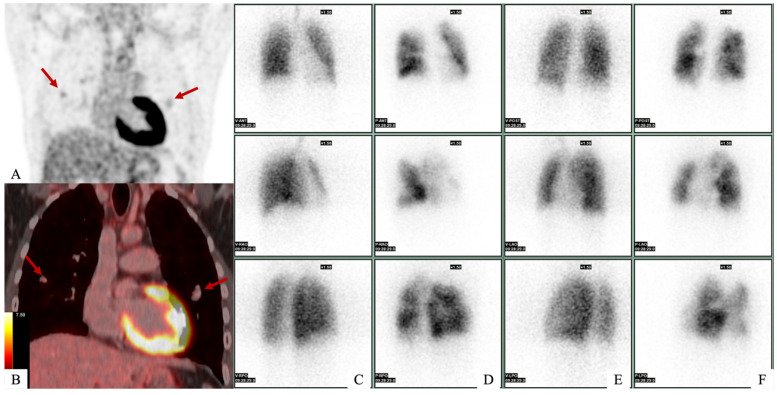
A 21-year-old man with no medical history presented to the emergency department with dyspnea and asthenia. Initial investigations included transthoracic echocardiography, which revealed a mass in the right atrium, and right heart catheterization, which confirmed pulmonary hypertension. Computed tomography (CT) confirmed the presence of a mass in the right atrium in contact with the tricuspid valve, as well as multiple bilateral pulmonary emboli and aneurysms. A complete work-up was carried out. (**A**,**B**) FDG-PET scan revealed mild intra-pulmonary hypermetabolism, which corresponded with pulmonary arterial aneurysms (*red arrows*), for which the SUVmax was measured at 2.4 with an aortic background noise of 2.6. (**C**–**F**) Ventilation/perfusion pulmonary scintigraphy revealed several bilateral perfusion defects on anterior (**D**) and posterior (**F**) views with systemized areas and other areas of hyperperfusion. There were no ventilation defects in both lungs on anterior (**C**) and posterior (**E**) view.

**Figure 2:**
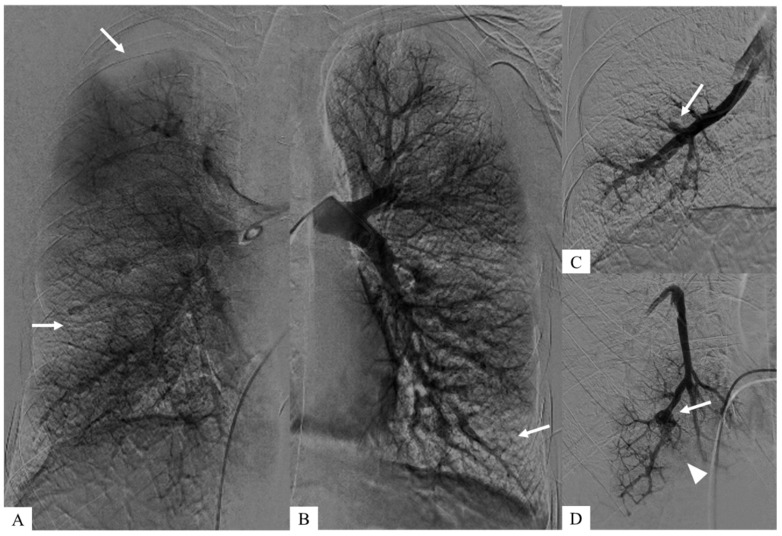
These aneurysms were identified during pulmonary angiography, enabling a more detailed vascular analysis and allowed right heart catheterization to confirm pre-capillary pulmonary hypertension. (**C**,**D**) Zoomed images after early intravascular injection show both pulmonary arterial aneurysms (*white arrows*) and downstream occlusions (*arrowhead*). (**A**,**B**) Coronal planar images of parenchymography, acquired at a later time after intravascular injection, show the consequences of pulmonary emboli corresponding to bilateral pulmonary defects (*white arrows*).

**Figure 3:**
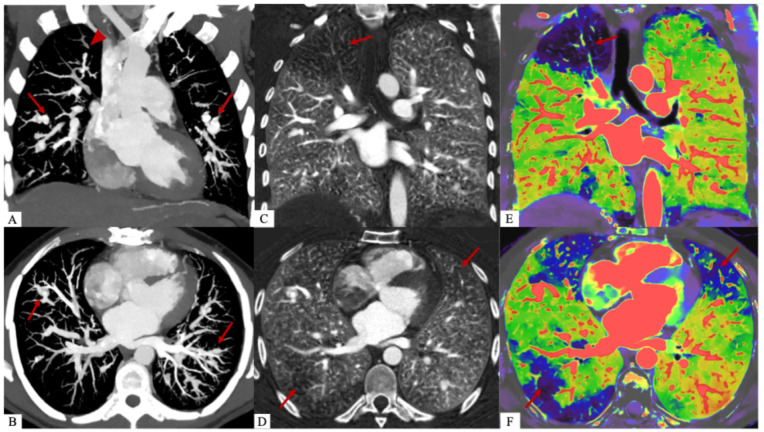
A dual-energy CT scan (Philips CT 7500, Philips Healthcare, Best, The Netherlands) was performed, showing that the pulmonary perfusion defects were consistent with areas of pulmonary embolism. It is also useful for distinguishing the different components, particularly the poorly enhanced myxomatous component from the purely vascular part of pulmonary arterial aneurysms. We calculated the extracellular volume (ECV) of myxomatous aneurysms at 43%, compared to 29% for healthy myocardium. This clearly shows an increase in the extracellular space due to the presence of the myxomatous matrix [1]. Dual-energy CT images with coronal (**A**) and axial (**B**) reconstructions at 40 keV and MIP after injection of contrast agent at the pulmonary arterial and aortic phase show bilateral pulmonary arterial vascular aneurysms (*red arrows*) and more hypodense intravascular myxomatous proliferations (*red arrowhead*). Coronal (**C**) and axial (**D**) lung iodine non-water maps show perfusion defects in territories of myxoma emboli (*red arrow*). Overlay coronal (**E**) and axial (**F**) images of the conventional and iodine density maps representing the same perfusion defects (*red arrows*).

**Figure 4:**
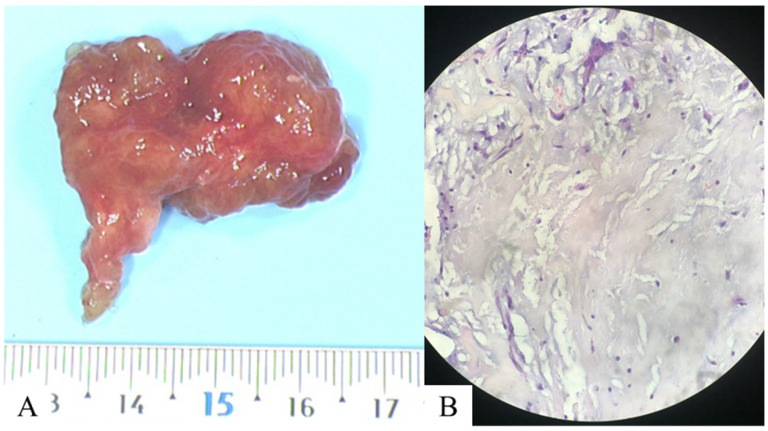
Following surgical resection of the atrial mass, a histopathological examination was performed, including (**A**) macroscopic, revealing a mass measuring approximately 4 × 3 × 1 cm, and (**B**) microscopic analyses on a slide after standard hematoxylin and eosin staining at 40× magnification. This revealed spindle-shaped star cells within an amorphous matrix, marked by anti-calretinin antibodies, which are consistent with a myxoma. Genetic analysis was negative. At the same time, a specific medical treatment for pulmonary hypertension was introduced (bosentan and sildenafil), which resulted in effective control of the condition and clinical improvement. The benefit of surgical treatment is not known, and medical treatment allowed a significant clinical improvement in our patient. Pulmonary hypertension secondary to the endovascular proliferation of myxoid cells is a rare condition [2]. Several mechanisms appear to be intertwined. This is due to the initial embolization of myxomatous cells, which adhere to the intima of pulmonary artery branches. The cells then proliferate, causing a more or less complete obstruction of the vessel. These cells can also cross the basement membrane and reach the media or adventitia, resulting in the formation of pulmonary arterial aneurysms [3]. The review of the iconography, in our case, shows all the different imaging modalities (non-invasive, nuclear medicine, and invasive), each of which has its advantages and disadvantages and, above all, the combined interest of the dual-energy CT. It allows for both a morphological and functional perfusion analysis, with the added possibility of quantifying the perfusion volume [4] and enabling a diagnosis of chronic thromboembolic pulmonary hypertension (CTEPH) [5]. It is also useful for distinguishing the different components, particularly the poorly enhanced myxomatous component from the purely vascular part of pulmonary arterial aneurysms. It allows all the different modalities to be combined into a single one [6]. Finally, it is worth noting the value of cardiac MRIs in diagnosing cardiac masses due to its high performance in tissue characterization, particularly in distinguishing between benign and malignant tumors [7].

## Data Availability

The data presented in this study are available on request from the corresponding author. The data are not publicly available due to containing confidential doctor and patient information.

## Abbreviations

The following abbreviations are used in this manuscript:

CT	Computed Tomography
FDG-PET scan	Fluorodeoxyglucose-Positron Emission Tomography
DECT	Dual-Energy CT
MIP	Maximum Intensity Projection
CTEPH	Chronic Tromboembolic Pulmonary Hypertension
